# Exploring Variability: Inflammation Mediator Levels across Tissues and Time in Poultry Experimentally Infected by the G1a and G6 Genogroups of Infectious Bursal Disease Virus (IBDV)

**DOI:** 10.3390/ani14111619

**Published:** 2024-05-29

**Authors:** Giovanni Franzo, Giorgia Dotto, Caterina Lupini, Matteo Legnardi, Claudia Maria Tucciarone, Francesca Poletto, Elena Catelli, Giulia Graziosi, Mattia Cecchinato, Daniela Pasotto

**Affiliations:** 1 Department of Animal Medicine, Production and Health, University of Padova, 35020 Legnaro, Italy; giorgia.dotto@unipd.it (G.D.); matteo.legnardi@unipd.it (M.L.); claudiamaria.tucciarone@unipd.it (C.M.T.); francesca.poletto.1@phd.unipd.it (F.P.); mattia.cecchinato@unipd.it (M.C.); daniela.pasotto@unipd.it (D.P.); 2 Department of Veterinary Medical Sciences, University of Bologna, 40064 Ozzano dell’Emilia, Italy; caterina.lupini@unibo.it (C.L.); elena.catelli@unibo.it (E.C.); giulia.graziosi2@unibo.it (G.G.)

**Keywords:** IBDV, gene expression, experimental infection, genogroups

## Abstract

**Simple Summary:**

Simple Summary: The infectious bursal disease virus (IBDV) is a major pathogen in the poultry industry due to its profound impact on the immune systems of young chickens. Various genetic types with distinct biological characteristics have been described. In this study, we evaluated the expression of different cytokines in the bursa of Fabricius and thymus over a 28-day experimental infection with two strains from the G1a (Classical) and G6 (ITA) genogroups. The G6 strain appeared to induce a more immediate immunosuppression, and unlike the G1a strain, did not show signs of gene expression recovery by the end of the study. This finding aligns with the higher replication level previously reported for the G6 and with the clinical outcome, as this genotype, although subclinical, has often been considered more immunosuppressive. Unlike other studies that focused on shorter periods post-infection, the patterns observed in this study were highly variable and complex, depending on the strain, tissue, and time point. Therefore, this study not only confirms the effect of strain/genogroup on immune system modulation but also highlights the importance of extended monitoring post-infection to better understand the intricate patterns and interactions with the host response.

**Abstract:**

Infectious bursal disease virus (IBDV) is a significant burden for poultry production and market due to both direct disease and induced immunosuppression. In the present study, the expression of different cytokines in the bursa of Fabricius and thymus was evaluated during a 28-day-long experimental infection with two strains classified in the G1a (Classical) and G6 (ITA) genogroups. Although both strains significantly affected and modulated the expression of different molecules, the G6 strain seemed to induce a delayed immune response or suppress it more promptly. A recovery in the expression of several mediators was observed in the G1a-infected group at the end of the study, but not in the G6 one, further supporting a more persistent immunosuppression. This evidence fits with the higher replication level previously reported for the G6 and with the clinical outcome, as this genotype, although subclinical, has often been considered more immunosuppressive. However, unlike other studies focused on shorter time periods after infection, the patterns observed in this paper were highly variable and complex, depending on the strain, tissue, and time point, and characterized by a non-negligible within-group variability. Besides confirming the strain/genogroup effect on immune system modulation, the present study suggests the usefulness of longer monitoring activities after experimental infection to better understand the complex patterns and interactions with the host response.

## 1. Introduction

Infectious bursal disease (IBD) is a highly contagious viral infection in poultry caused by the infectious bursal disease virus (IBDV). This disease significantly impacts the global poultry industry due to its profound effect on the immune system of young chickens, leading to severe immunosuppression and increased susceptibility to secondary infections [1,2].

IBDV is classified under the species *Avibirnavirus gumboroense*, within the genus *Avibirnavirus*, and belongs to the family *Birnaviridae* (https://ictv.global/taxonomy accessed on 12 March 2024). The virus is characterized by a non-enveloped virion and a double-stranded RNA genome comprising two segments, named A and B. Segment A, which is 3.2 kb long, codes for a capsid protein (VP2), a scaffold protein (VP3), a protease (VP4), and a non-structural protein with regulatory and anti-apoptotic functions (VP5). Segment B, 2.9 kb in length, encodes the RNA-dependent RNA polymerase [3,4]. Of the two known serotypes of IBDV, only the serotype 1 is pathogenic.

Like other RNA viruses, IBDV exhibits a high mutation rate, leading to the emergence of several variants with distinct genetic, antigenic, and biological characteristics [1,5,6]. The “classical” strains of IBDV were first reported in the USA in the late 1950s [7]. In the 1980s, two more types of IBDV were identified: one included highly pathogenic strains (termed “very virulent”) found across Europe, Africa, and Asia, while the other comprised “variant” strains, antigenically distinct from other IBDVs, primarily circulating in North America [8]. These variant strains eventually spread to Eastern Asia in the 2010s [9].

More objective, unified classification approaches based on genetic sequence analysis have been proposed in recent years. Various classification systems, relying on phylogeny either based on part of the VP2 [10] or both VP2 and VP1 genes [3,11], have been instrumental for strain characterization, retaining the information provided by traditional classification whilst allowing to identify multiple novel genetic types.

Among these is the so-called ITA genotype, first detected in 2011 in IBD-live vaccinated Italian broilers [12]. A recent classification placed the ITA genotype into genogroup 6 (G6) [10]. Full genome characterization revealed that ITA-IBDV possesses unique molecular characteristics at key positions, potentially leading to significant changes in virus properties [13]. The experimental infection of specific-pathogen-free (SPF) chickens with an ITA genotype strain highlighted its aggressiveness towards lymphoid tissues, affecting not only the bursa of Fabricius, despite a subclinical course [14]. Following their identification in Italy, G6 strains were also reported in Middle Eastern countries as well [10,15], further substantiating their epidemiological relevance.

Primary viremia, occurring through portal circulation, introduces IBDV into bursal follicles, where extensive replication occurs in B lymphocytes [2,16,17]. Specifically, IgM+ B cells are the primary targets of IBDV, with surface immunoglobulin M (sIgM) identified as the cellular receptor [1,18]. Very virulent IBDV (vvIBDV) strains are known to cause severe lesions in non-bursal lymphoid organs, such as the thymus, spleen, and cecal tonsils, likely due to the virus action in these areas [19,20]. B lymphocyte depletion is largely attributed to cell lysis and apoptosis [21,22]. However, a critical aspect of IBDV interaction with the host involves its modulation of cytokine expression [23,24,25,26,27]. Cytokines, essential signaling molecules in the immune system, play pivotal roles in initiating and regulating immune responses. IBDV pathogenesis involves a complex interplay with the host’s cytokine network [28,29]. For example, vvIBDV strains are known to induce a strong pro-inflammatory cytokine response, including increased levels of IFN-γ, IL-6, and TNF-α, likely contributing to severe bursal tissue damage and immunosuppression [2,18,25,27]. Conversely, anti-inflammatory cytokines like IL-10 may be upregulated to counteract this inflammation, further complicating the immune response [25,30].

This study experimentally evaluates the difference in the expression profile of cytokine and other inflammation and apoptosis mediators, between the ITA-IBDV (G6) genotype and a “classical” genogroup 1a strain. The study also assesses the consequences in terms of viral titers, serological responses, and tissue damage.

## 2. Materials and Methods

### 2.1. Samples

Samples were obtained within the context of two previous studies, namely Lupini et al. [14] and Silvera et al. [20]. Briefly, SPF chickens were utilized and housed in HEPA-filtered isolators for the duration of the study, with food and water provided ad libitum. The trial was conducted under national regulations on animal experiments and welfare, authorization number 635/2015-PR, granted by the Italian Ministry of Health. This study used two isolates of IBDV, named according to the nomenclature proposed by Jackwood et al. [31]: IBDV 1/chicken/Italy/1829/11/(G6) (ITA genotype, genogroup 6) and IBDV 1/chicken/Italy/24II/12/(G1a) (genogroup 1a, grouping classical strains), which was used for comparative purposes. At hatching, birds were divided into three groups: the G6-IBDV group (30 birds), the G1a-IBDV group (30 birds), and the controls (18 birds), each housed in separate poultry isolators. At the age of 35 days, the G6-IBDV and G1a-IBDV groups were orally inoculated with 10^4.5^ EID50 of the IBDV strains 1/Italy/1829/11/(G6) and 1/Italy/24II/12/(G1a), respectively. The control group was mock-inoculated. At 2, 4, 7, 14, 21, and 28 days post-inoculation (DPI), five birds from both the G6-IBDV and G1a-IBDV groups and three from the control group were humanely euthanized. Tissue samples from the bursa of Fabricius and thymus were aseptically collected and stored. For a more detailed description, we refer to the original publications [14,20].

### 2.2. RNA Extraction and Reverse Transcription

Tissue samples were weighed and mechanically homogenated in a 10% suspension weight/volume PBS solution using T10 basic ULTRA-TURRAX^®^ (IKA^®^-Werke GmbH & Co. KG, Staufen, Germany) supplied with sterile disposable plastic probes to prevent sample-to-sample cross-contamination. The homogenized tissues were centrifuged at 2000 rpm for 15 min at +4 °C and the supernatant was collected. RNA was extracted using the High Pure RNA Isolation Kit (Roche Diagnostic, Marnes La Coquette, France), according to the manufacturer’s instructions. cDNA was produced using random primers starting from 5 µL of RNA using the Maxima™ H Minus cDNA Synthesis Master Mix kit (Thermo Fisher Scientific, Waltham, MA, USA). To remove any residual DNA contamination, a DNase incubation step, provided by the kit, was included. cDNA was stored at +4 °C and relative quantification was performed within one hour.

### 2.3. Relative Quantification Assay Validation

The expression of different genes was assessed by relative quantification and comparison with housekeeping genes using the ∆Ct method. Primers (Table 1) were selected from the literature and internally validated.

All the reactions were performed using the PowerUp™ SYBR™ Green Master Mix kit (Thermo Fisher Scientific, Waltham, MA, USA) on a LightCycler95 instrument (Roche, Basel, Switzerland).

Different primer concentrations and thermal protocols were evaluated by performing and testing 10-fold RNA dilution curves obtained from a bursal sample. Reverse transcription was performed as previously described.

The optimized protocol was finally validated by testing the dilution curves in triplicate on three different days to assess the repeatability of the test and calculate the efficiency of each reaction, necessary for relative quantification. The absence of non-specific amplification was verified by performing a melting curve analysis.

### 2.4. Relative Quantification

For each sample, all genes were simultaneously tested in the same run according to the following protocol. A total of 2 µL of cDNA were added to a standard mix including 1X PowerUp™ SYBR™ Green Master Mix and 0.8 µL of each primer and biology-grade water, up to a final volume of 10 µL. The thermal protocol included a polymerase activation step at 95 °C for 2 min, followed by 45 cycles at 95 °C for 15 s, 55 °C for 15 s, and 72 °C for 1 min. After the last extension, a melting curve analysis was performed by progressively increasing the temperature (ramp rate: 5 0.1 °C/s) from 40 °C to 90 °C and continuously monitoring the fluorescence data. The previously determined reaction efficiency was included for each gene to calculate efficiency-corrected Cq values, which allowed to analyze the relative gene expression calculating the ratio between the target gene and Actin and GADPH, selected as housekeeping genes.

### 2.5. Statistical Analysis

The ratio of each gene expression among groups was compared within tissue and day using the non-parametric Kruskal–Wallis test, followed by post hoc test of Mann–Whitney test with Bonferroni correction for multiple comparisons. All analyses were performed in R. The statistical significance level was set at *p* < 0.05. Graphics were generated with the R libraries *ggplot2* and *ggpubr* [33,34].

## 3. Results

### 3.1. Clinical Pathological and Virological Outcomes

IBDV detection and lesion evaluation was performed in previous studies [14,20]. However, to enhance the understanding of the overall results, the main findings are summarized in this paper. For a more detailed description, we refer to the original publications.

### 3.2. IBDV Detection

No statistically significant differences were observed in the number of IBDV-positive birds across different sampling days. However, when examining viral RNA quantification in bursal tissues, notable differences emerged at specific time points. Specifically, at 4 and 7 days post-inoculation (DPI), the mean viral RNA load in the G6-IBDV group was significantly higher than in the G1a-IBDV group (*p* < 0.05)

A significantly higher prevalence of IBDV-positive birds in the thymus was recorded in the G6-IBDV group at 14 DPI when compared to the G1a-IBDV group (*p* < 0.05). Additionally, the G6-IBDV group demonstrated a consistently higher RNA load in the thymus at 2, 4, 7, and 14 DPI, significantly surpassing the levels observed in the G1a-IBDV group (*p* < 0.05).

### 3.3. Lesions

No significant differences in the bursa-to-body weight ratio (B/BW) were observed with the only exception of day 4, when a significantly smaller weight ratio was observed in the G6-IBDV, (*p* < 0.05). More severe histological lesions were detected in the G6-infected group at day 2 (*p* < 0.05), while, although higher scores were observed at day 4 and 7, the statistical significance was not reached at other time points.

In the thymus, no differences in the thymus-to-body weight ratio (T/BW) and in the severity of the histological lesions were observed during the whole study period.

### 3.4. Gene Expression

Different groups exhibited variable gene expression patterns depending on the DPI, evaluated mRNA, tissue types, and infection strains, in addition to significant variability among individuals. However, some trends could still be identified. For a more detailed report of the statistically significant differences, we refer to Supplementary Figures S1 and S2.

#### 3.4.1. Bursa of Fabricius

BAX was overall more expressed in the infected groups, with an earlier increase in G1a at DPI 2 and 4, and at the end of the study, while G6 caused higher expression levels between DPI 7 and 21. However, significant differences were observed at DPI 2, 14, and 28 only. G1a induced an overall higher BCL2 level for the whole study duration, with DPI 4 being the only, not significant, exception. The IBDV infection caused, in both groups, a higher expression of CASP-9, which was more prominent in the G6 group, particularly after DPI 14, peaking at DPI 21 and decreasing thereafter.

An overall overlapping pattern could be identified for IFN-β, IFN-γ, IL-1β, and IL-6, featured by a sharp increase at 2 DPI in both infected groups, and thereafter by a marked decrease. However, significant differences, albeit of a lower magnitude, were detected at different DPI. IFN-β was significantly higher for G1a at DPI 2, 21 and 28, while higher values were observed at DPI 7 in the G6-group. IFN-γ was more expressed in G1a compared to controls only at DPI 2. IL-1β was overexpressed in the G6 group at DPI 2, 4, and 7, and on DPI 21 and 28 in G1a, even if significant differences were detected at DPI 2 and 21. A similar pattern, with an overall earlier higher expression in the G6, and a delay in the G1a occurred for IL-6 also, even if a statistical difference was reached at DPI 2 only. G1a induced a higher expression of IL-10 for the whole study duration, especially until DPI 7, when the differences were statistically significant. Similarly, IL-2 was more expressed in G1a compared to G6 (which was even slightly under-expressed compared to controls), followed by depression at DPI 14 and rebound thereafter. IL-4 was overall under-expressed for the whole considered period in the G6 group, while a normal or higher expression featured G1a until DPI 7, when a significant decrease in expression level was identified also in this group. An under-expression of IL-5 featured the G6 until DPI 4 and at DPI 21 compared to G1a, while the scenario was reversed at DPI 14, when IL-5 was significantly less expressed in G1a.

**Figure 1:**
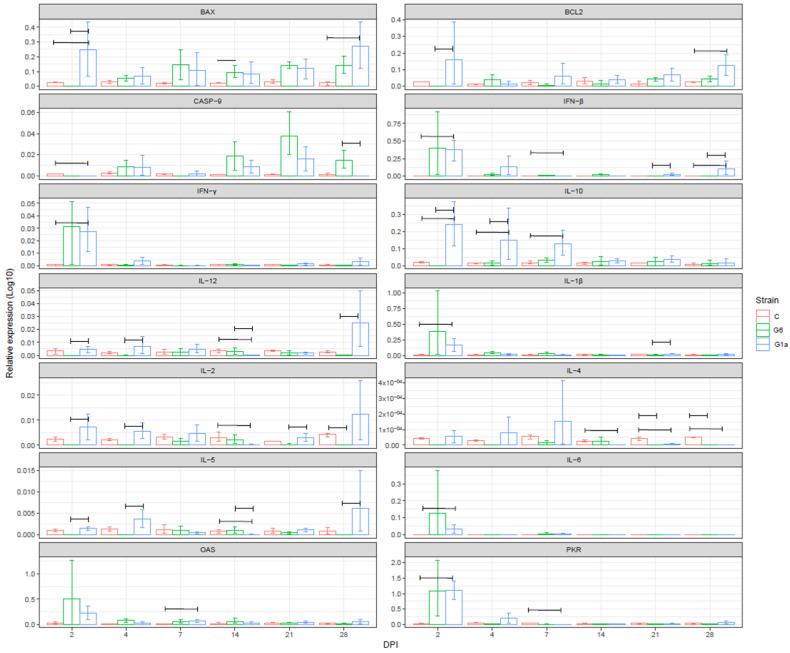
Bar plot depicting the average and 95% confidence interval (CI) of relative gene expression in the bursa of Fabricius (presented on a log10 scale) of the experimental groups at different days post-infection (DPI). The genes under consideration are shown in separate windows. Group pairs featured by the statistically significant differences that were detected are linked by a bracket.

A remarkable increase in OAS and PKR expression was detected at DPI 2, followed by a rapid decline leading to an expression level comparable to the controls at about DPI 7 (Figure 1).

#### 3.4.2. Thymus

A higher BAX expression level was observed in the G6 group compared to the controls at DPI 2 and 4. Greater expression levels in G1a were also observed as well, although the high within-group variability impeded the achievement of statistical significance until 7 DPI. BCL2 was less expressed compared to the control groups, especially at the end of the study (after DPI 21). CASP-9 was on average low and comparable to the control group, except on DPI 21, when G1a revealed a higher expression compared to both G1a and controls, although also at this time point, the average ratio values were overall low. The expressions of IFN-β and IFN-γ were lower than the controls throughout the study period, despite the differences being significant at limited DPI only. The same holds true for IL-1β, IL-4, and IL-6, with few exceptions at specific DPI when higher average values characterized the infected groups, but featured high among-subjects variability.

**Figure 2:**
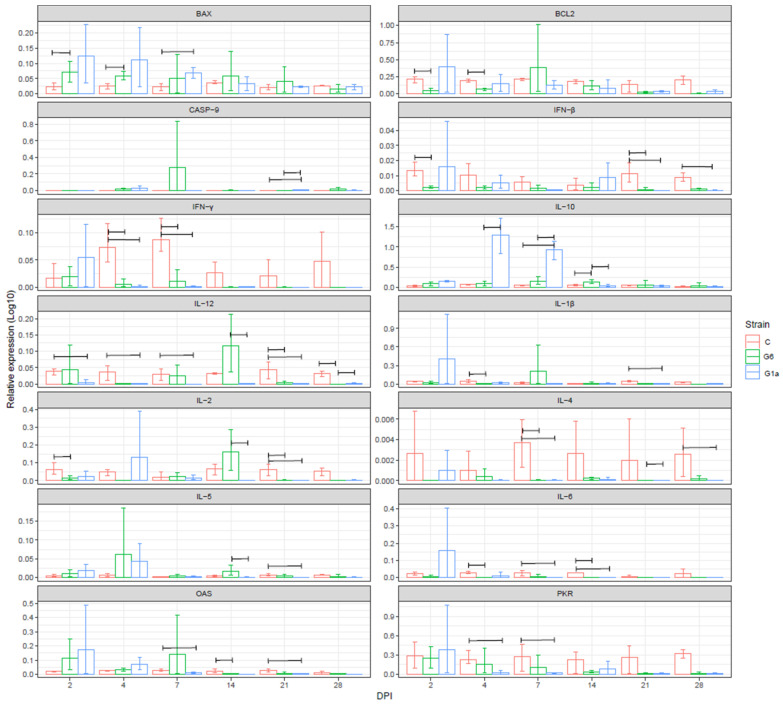
Bar plot depicting the average and 95% confidence interval (CI) of relative gene expression in the thymus (presented on a log10 scale) of the experimental groups at different days post-infection (DPI). The genes under consideration are shown in separate windows. Group pairs featured by the statistically significant differences that were detected are linked by a bracket.

A more variable pattern was observed for IL-12, being under-expressed in G1a, while G6 demonstrated an expression level comparable to the control until DPI 14 and under-expression thereafter. No major differences in IL-5 expression were reported until DPI 14, when the G1a-infected group level decreased below the G6 (DPI 14) and control (DPI 21) groups. OAS expression levels were highly variable in the infected groups, and a significant increase was observed at DPI 7 in the G6 group only, followed by a decrease below the control levels in both infected groups. PKR was comparable or less expressed in the IBDV-infected groups for the whole study duration (Figure 2).

## 4. Discussion

IBDV pathogenesis largely depends on its interactions with the immune system, causing direct damage and affecting its ability to respond to external stimuli. Different genotypes and strains exhibit varying virulence, lesion severity, and persistence. Variable interactions with the host, as reported in previous studies, can also be proposed [1]. Lupini et al. [14] and Silveira et al. [20] demonstrated the immunopathogenicity of G6 strains, despite the subclinical course and differential behavior compared to strains classified as G1a. Our study delved deeper into this topic, revealing variable interactions and interference with the expression of different cytokines. One of our main findings was the complexity of gene expression patterns, which were often heterogeneous over time and among individuals. Outliers were processed and tested several times, leading to consistent results, which supports the reliability of the developed assays and the presence of biological differences among individuals, even within the same genetic line. This variability often hindered the ability to achieve statistically significant differences among groups. However, trends were still present and could be further investigated.

In the bursa, CASP-9 induction, an initiator caspase that plays a key role in the intrinsic pathway of apoptosis by activating downstream effector caspases, occurred in both groups, although it was more pronounced in the G6-infected group, especially after 14 DPI, suggesting a direct effect of this mechanism on lymphocyte depletion, similar to the observations by Huang et al. for vvIBDV [27]. However, the upregulation in that study was of a higher magnitude and peaked at about 5 DPI, while in our study, a more delayed peak (about 21 DPI), with lower but persistent upregulation, was observed and likely was involved in the markedly different clinical course and histologic lesions caused by vvIBDV strains. The levels of the pro-apoptotic gene BAX were higher in the infected groups, with a slightly higher expression in G6 in the range of 7–21 DPI that might explain the different induction of the caspase system between the two genogroups considered in this study, combined with the decrease in the anti-apoptotic BCL2 expression (not observed in G1a).

The different study length compared to that of Huang et al. prevents a proper comparison with vvIBDV strain behavior. However, a more persistent infection, characterized by less severe signs, may be at least partially justified by our findings. Notably, higher viral titers were previously demonstrated in the bursa of subjects infected with G6 compared to G1a until 14 DPI [20]. Since the increase in apoptotic mediators followed, rather than preceded, the higher viremia levels, the induction of the apoptotic system might be the consequence, rather than the cause, of such viral load, although implications for immunosuppression persistence can reasonably be hypothesized. Conversely, an even more precocious induction (2 DPI) of pro-inflammatory molecules was observed in both the G1a and G6 groups compared to what was observed by Huang et al. Thereafter, an extremely rapid decline reached the levels of controls at just 4 DPI and persisted until the end of the study, although with a tendency for recovery at 28 DPI in the G1a-infected group. In G6, IL-2, IL-12, IL-4, and IL-5, involved in the growth, proliferation, and differentiation of Th1 and Th2 cells, were notably less expressed in the early days post-infection compared to G1a, where an expression level comparable to or higher than the controls was observed. Interestingly, vvIBDV strains were characterized by a significant increase in such mRNA expression [25], although some studies reported the extremely precocious (i.e., first hours post-infection) downregulation of most pro-inflammatory molecules, followed by a rebound and fluctuations thereafter [26]. In fact, the overexpression of pro-inflammatory cytokines, as demonstrated in vvIBDV strains, can lead to a cytokine storm contributing to viral pathogenesis, although potentially involved also in viral clearance [29]. Such strong immune system activation was not observed in the strains investigated in our study, where immunosuppression dominated. Similarly, Rautenschlein et al. (2003) reported a marked depression of several inflammation mediators, with significant variability depending on the considered strain, but only virulent ones were still able to induce IL-2 expression at 5 DPI [19]. This difference might explain the clinical courses, with a cytokine storm induced by vvIBDV involved in the pathogenesis and severity of lesions. Conversely, the lower activation or suppression of inflammatory mediators observed in this study could protect from the most serious direct damages but allow for a longer viral replication and persistence, as also suggested by the higher viral titer of G6 compared to G1a [20]. The persistent inhibition or downregulation of antiviral molecules, like OAS and PKR antiviral proteins that inhibit protein synthesis or promote viral RNA cleavage through RNase L activation, probably contributed to viral persistence. Chen et al. [25] reported the induction of these molecules as early as 2–4 DPI [25]. Such an early induction was confirmed in our study, but transcription was thereafter consistently inhibited by both viruses. This evidence, true for other mediators as well, underscores the need to extend monitoring beyond the first days post-infection, as the evolution of expression profiles over time can be complex and challenging to interpret, especially due to the high variability among subjects expected over longer periods. One of the main limitations in this regard is the impossibility of sampling the same subjects over time, making it difficult to disentangle the sources of variability. However, with limited exceptions, the results are consistent within groups, allowing at least the investigation of overall trends.

The detection of a proinflammatory increase at 2 DPI, as opposed to the depression observed in some vvIBDV strains, might also testify to the ability of vvIBDV strains to rapidly inhibit the immune response, allowing a significant replication in the early infection stages and leading, in turn, to a rebound activation and cytokine storm. The quicker response observed for less virulent strains could prevent this damaging mechanism. Nevertheless, the early activation was still ineffective or circumvented by other mechanisms in the long term, leading to viral persistence and immune system depression.

Interestingly, IL-10, which has a negative feedback regulation on the immune system [27,35], was upregulated in G1a, which contrasts with the higher depression of the immune response induced by G6. Therefore, more complex mechanisms of immunosuppression could be hypothesized. Conversely, the higher induction of this molecule might contribute to alleviating more severe tissue damages [24].

Silvera et al. [20] reported an even more marked difference in viral replication between G1a and G6 in the thymus, although also in this case, only limited and transient differences in lesion severity were reported. A different BAX and BCL2 induction compared to the controls emerged in the earliest days of the study, while a higher caspase induction was observed at 21 DPI. The inhibition of the molecules involved in antiviral response and antigen-presenting cell activation, like INF-β and INF-γ, as well as lymphocyte differentiation promoters, such as IL-4 and IL-6, was even more evident than in the bursa and affected especially the first DPI in the G6-infected group. Such a delayed activation of the immune response, rather than apoptotic-related mechanisms, might thus explain the higher viral replication of this genogroup. A higher activation of IL-10 was also reported in G1a in the thymus, which could confirm the limited contribution of IL-10 overexpression in supporting IBDV replication. No activation or even downregulation of antiviral genes was present, especially for PKR, with only a transient activation at 2 DPI of OAS, more evident in G1a, further contributing to explaining the different replication levels. Accordingly, Chen et al. [25] reported the higher induction of PKR caused by vvIBDV compared to attenuated strains, while a more prominent OAS induction was associated with the B87 vaccine. If this might be associated with differential virulence and persistence, and thus can be extended to the present study results, will need further investigations.

## 5. Conclusions

The present study investigates the impact of strains belonging to different genogroups on the induction/inhibition of the expression of inflammation mediators. Most of the previous studies reported an upregulation of pro-inflammatory molecules, especially in strains characterized by a higher virulence. However, those studies were often limited to the first hours or days after infection. While our study largely agreed with previous findings when earlier DPI were considered, much more complex and less evident and predictable differences were observed in later infection stages, with some mediators increasing or decreasing depending on the interaction among strain, tissue, and infection stage. Overall, a higher, although transient, immune activation was initially observed in the G1a-infected group compared to G6. Additionally, the evidence of immune system reactivation at 28 DPI was detected for several molecules in the G1a group, whose level showed a trend toward an increased expression. Such evidence could demonstrate a more efficient response and prompt recovery of the immune system functionality. Such a pattern may fit with the longer viral persistence and relevant immunosuppression often described for G6, even in the absence of overt clinical signs. The variable cytokine patterns observed in the bursa and thymus might be due to their different immunological roles and involvement in the IBDV pathogenesis.

Although no conclusive evidence can be provided, we believe that the extension of immune functionality evaluation for longer periods during experimental infections would be useful for a proper understanding of the pathogenic mechanisms and comparison of IBDV with different virulence levels, shedding light on the complex interaction among inflammatory mediators and their dysregulation.

## Figures and Tables

**Table 1:** List of primers used in the present study.

Primer	Sequence	Reference
ACTIN-F	TGCTGTGTTCCCATCTATCG	[27]
ACTIN-R	TTGGTGACAATACCGTGTTCA	[27]
GADPH-F	TGCTGCCCAGAACATCATCC	[27]
GADPH-R	ACGGCAGGTCAGGTCAACAA	[27]
BAX-F	GTGATGGCATGGGACATAGCTC	[27]
BAX-R	TGGCGTAGACCTTGCGGATAA	[27]
BCL2-F	ATCGTCGCCTTCTTCGAGTT	[27]
BCL2-R	ATCCCATCCTCCGTTGTCCT	[27]
CASP-9-F	CCGAAGGAGCAAGCACG	[27]
CASP-9-R	AGGTTGGACTGGGATGGAC	[27]
IFN-B-F	TTCTCCTGCAACCATCTTC	[25]
IFN-B-R	GAGGTGGAGCCGTATTCT	[25]
IFN-G-F	ATCATACTGAGCCAGATTGTTTCG	[27]
IFN-G-R	TCTTTCACCTTCTTCACGCCAT	[27]
IL-10-F	AGCTGAGGGTGAAGTTTGAGGAA	[27]
IL-10-R	CAGGACCTCATCTGTGTAGAAGCG	[27]
IL-12-F	AGGTGGGTCTGGCTTT	[27]
IL-12-R	TTCTGAGACTGGTGGCTTCACTTCC	[27]
IL-1B-F	GCTCTACATGTCGTGTGTGATGAG	[27]
IL-1B-R	TGTCGATGTCCCGCATGA	[27]
IL-2-F	TTCTGGGACCACTGTATGCTCTT	[32]
IL-2-R	TACCGACAAAGTGAGAATCAATCAG	[32]
IL-4-F	GCTCTTATGCAAAGCCTCCACAA	[27]
IL-4-R	TGCTGCTGGCATTCAGGAGC	[27]
IL-5-F	GGAACGGCACTGTTGAAAAATAA	[32]
IL-5-R	TTCTCCCTCTCCTGTCAGTTGTG	[32]
IL-6-F	GCTCGCCGGCTTCGA	[27]
IL-6-R	GGTAGGTCTGAAAGGCGAACAG	[27]
OAS-F	CACGGCCTCTTCTACGACA	[25]
OAS-R	TGGGCCATACGGTGTAGACT	[25]
PKR-F	CCTCTGCTGGCCTTACTGTCA	[25]
PKR-R	AAGAGAGGCAGAAGGAATAATTTGCC	[25]

## Data Availability

The original contributions presented in the study are included in the article and Supplementary Material, further inquiries can be directed to the corresponding authors.

## Supplementary Materials

The following supporting information can be downloaded at: https://www.mdpi.com/article/10.3390/ani14111619/s1. Supplementary Figure S1. Bar plot illustrating the average and 95% confidence interval (CI) of the relative gene expression in the bursa of Fabricius (expressed on a log10 scale) of the experimental groups. Different genes and days post-infection (DPI) are represented in separate rows and columns, respectively. An independent *y*-axis scale was employed for each plot to enhance the visibility of differences among groups for each DPI. Statistical significant differences are coded (i.e., *: *p* ≤ 0.05; **: *p* ≤ 0.01; ***: *p* ≤ 0.001) under the corresponding bracket. Supplementary Figure S2. Bar plot illustrating the average and 95% confidence interval (CI) of the relative gene expression (expressed on a log10 scale) for various groups in the thymus. Different genes and days post-infection (DPIs) are represented in separate rows and columns, respectively. An independent *y*-axis scale was employed for each plot to enhance the visibility of differences among groups for each DPI. Statistical significant differences are coded (i.e., *: *p* ≤ 0.05; **: *p* ≤ 0.01; ***: *p* ≤ 0.001) under the corresponding bracket.
