# Supplementary figures and images for: Exploring Variability: Inflammation Mediator Levels across Tissues and Time in Poultry Experimentally Infected by the G1a and G6 Genogroups of Infectious Bursal Disease Virus (IBDV)
Source: Animals (Basel). 2024 May 29;14(11):1619. doi: 10.3390/ani14111619 (PMC11171315; doi:10.3390/ani14111619)

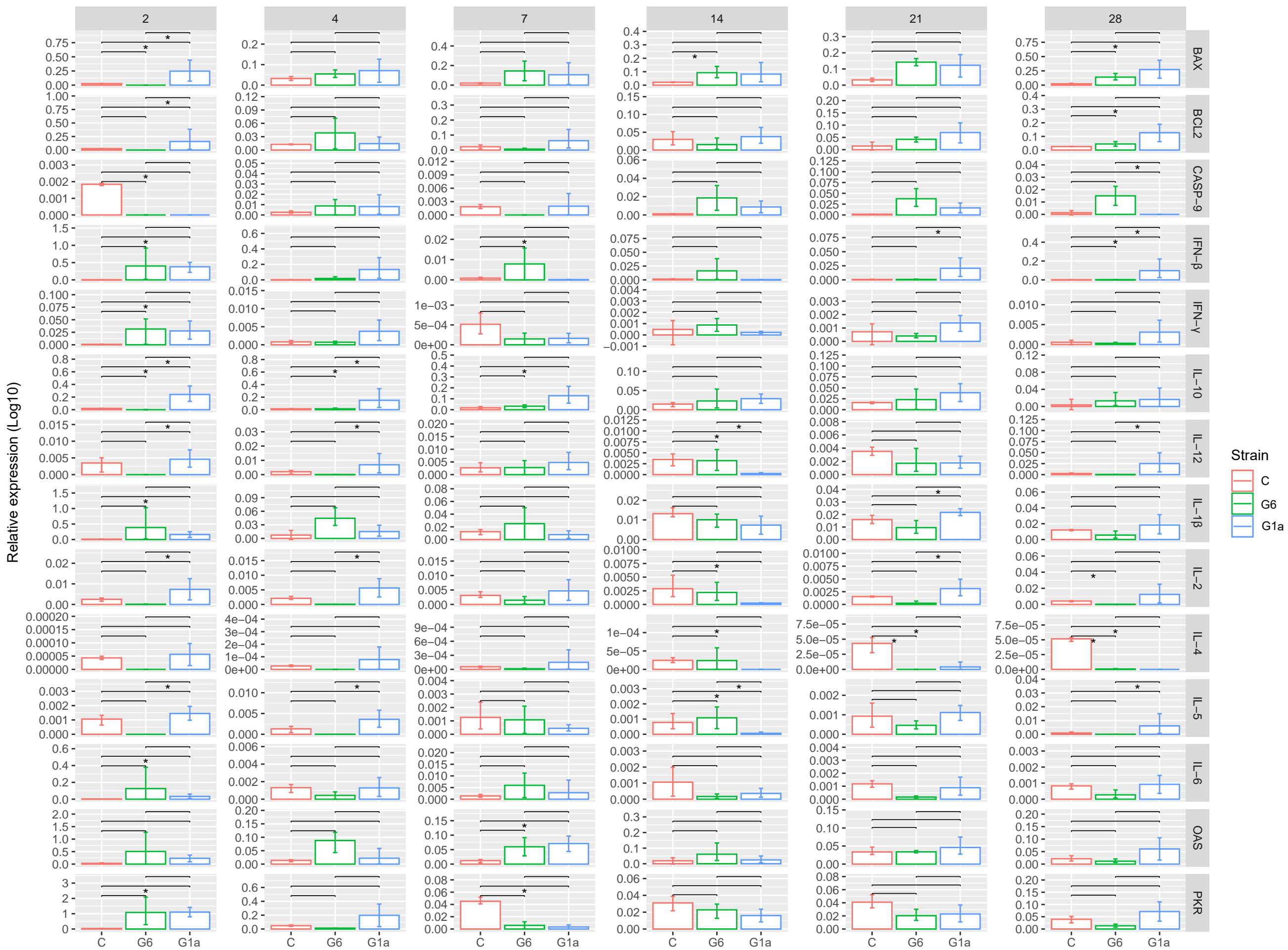

Supplement: Supplementary file 1 [file animals-14-01619-s001.zip › Supplementary figure S1.pdf]

Relative expression (Log10)

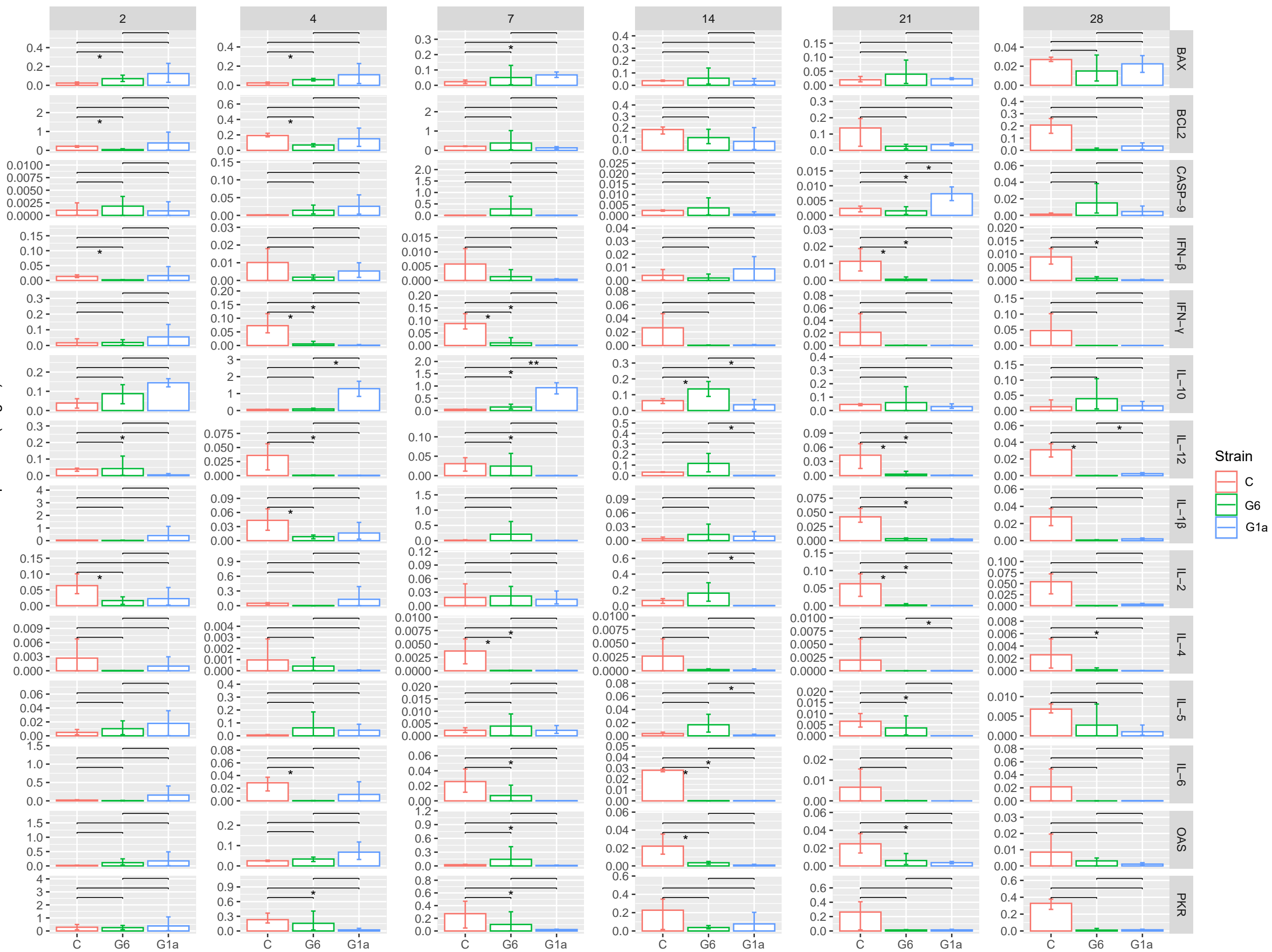

Supplement: Supplementary file 1 [file animals-14-01619-s001.zip › Supplementary figure S2.pdf]
